# An Adult Patient With Posterior Unilateral Crossbite Treated by Corticotomy‐Assisted Expansion

**DOI:** 10.1155/crid/9744364

**Published:** 2025-12-14

**Authors:** Ahmad A. Al-Fraidi, Baker M. Sedqi, Rana S. Alamri

**Affiliations:** ^1^ Ministry of Health, Department of Orthodontics, King Fahad Hospital, Specialized Dental Center, Madina, Saudi Arabia, kfmc.med.sa; ^2^ Ministry of Health, Department of Periodontics, Ohud Hospital, Specialized Dental Center, Madina, Saudi Arabia, ohudhospital.com

**Keywords:** corticotomy, expansion, transverse deficiency, unilateral crossbite

## Abstract

Maxillary transverse deficiency is a common clinical problem in orthodontics, and treating it becomes more challenging when a unilateral posterior crossbite exists. Different treatment modalities have been used, including slow maxillary expansion (SME), rapid maxillary expansion (RME), surgically assisted rapid palatal expansion (SARPE), and corticotomy‐assisted expansion. The type of treatment depends mainly on the patient′s skeletal maturity. In this case report, we describe the treatment of a 20‐year‐old female patient with unilateral posterior crossbite using corticotomy‐assisted expansion with fixed orthodontic appliances. The crossbite was corrected, and normal overbite and overjet were achieved with Class I canine and molar relationships. The duration of correction of the crossbite was relatively shorter, and periodontal health was maintained without any complications. The technique of selective alveolar bone corticotomy is considered a less invasive procedure when compared to SARPE and MARPE, and it may provide an efficient treatment option in cases of adult unilateral maxillary transverse deficiency.

## 1. Introduction

Maxillary transverse deficiency is a common clinical problem for orthodontists in establishing ideal alignment and occlusion [1].

This case is more challenging when a unilateral posterior crossbite exists. The prevalence of unilateral posterior crossbite varies from 8% to 23% [2]. Different treatment modalities have been used, including slow maxillary expansion (SME), rapid maxillary expansion (RME), surgically assisted rapid palatal expansion (SARPE), miniscrew‐assisted rapid palatal expansion (MARPE), and corticotomy‐assisted expansion. The type of treatment depends mainly on the skeletal maturity [3].

For long‐term effective changes and to exhibit significant changes at the skeletal level in both the circummaxillary and maxillary structures, treatment preferably starts before the pubertal peak of the growth spurt. When RME treatment is performed after the pubertal growth spurt, adaptations of maxillary expansion are more dentoalveolar than those at the skeletal level [3].

SME and RME are successful in growing patients. In contrast, in skeletally mature patients, the conventional approach of slow expansion could be problematic, limited, and inefficient, as it might compromise periodontal health if performed beyond a few millimeters [2].

Routine orthodontic expansion treatment of a unilateral posterior crossbite in adults might be problematic as orthodontic appliances exert bilateral effects when activated, resulting in overexpansion on the nonaffected side. This is not an uncommon complication in unilateral crossbite cases, which require extra effort and time for management. Although different modifications have been made to orthodontic appliances to minimize the bilateral effect, it always exists.

The aim of the treatment of true unilateral posterior crossbite is to move selected teeth on the constricted side of the maxillary arch. Conventional expansion appliances used to treat true unilateral posterior crossbite might be problematic, as the maxillary dental arch will expand bilaterally, resulting in undesirable overexpansion of the unaffected side. [4] In this situation, overcorrection on the unaffected side is a common consequence that may lengthen the overall treatment time and make it difficult to correct [2].

In adult orthodontic treatment, corticotomy‐assisted orthodontic treatment is a promising technique with various applications, as it assists in overcoming many of the limitations of the treatment, such as its long duration and possibility for periodontal issues [5]. Köle [4] first reported the use of corticotomy in orthodontics in 1959, followed by Converse and Horowitz [6] in 1969. Lines [7] also reported maxillary expansion accompanied by corticotomy in 1975. Wilcko et al. [8–10] reintroduced the concept in 2001 and named the patented technique “accelerated osteogenic orthodontics” (AOO). It is also called “periodontally accelerated osteogenic orthodontics” (PAOO).

This technique combines the “bone activation” (selective alveolar decortication, osteotomies, bone thinning, and alveolar augmentation using particulate bone grafting material) and orthodontic treatment [8]. The regional accelerating phenomenon (RAP) uses alveolar bone decortication to increase regional bone remodeling to accelerate tooth movement and ridge augmentation procedures to overcome the deficiency of the alveolar ridge. To increase the limits of tooth movement, decrease treatment times, and facilitate rapid recovery of unerupted teeth [8], and to be more effective for molar uprighting [11], these potential advantages make AOO treatment more effective in comparison with traditional orthodontics. AOO is a good alternative to conventional orthodontic mechanics for the treatment of true unilateral crossbite in adult patients, and conventional orthodontic mechanics are either less efficient, patient dependent, or accompanied by side effects, such as overexpansion on the normal side, compromised vertical dimension, and canting of the occlusal plane. The minimally invasive cortectomy technique, piezocision, can be used as an adjunct for orthodontic treatment in adult patients. However, some complications have been reported, such as gingival clefts and injury to the root and mental foramen [5]. Corticotomy‐assisted orthodontics has been advocated for the management of UPBC [2]. The proposed mechanism of action is that when corticotomy is performed on the affected side, resistance to tooth movement is reduced, and teeth move faster than on the nonaffected side [2]. This case report describes the treatment of a 20‐year‐old female having a unilateral posterior crossbite using corticotomy‐assisted orthodontics.

## 2. Case

### 2.1. History and Diagnosis

A 20‐year‐old female patient visited the orthodontic clinic with a chief complaint of “My bite on the right side is not like the left side.” Her medical and dental history was not significant. The temporomandibular joint was healthy. The patient was periodontally healthy. Extraoral examination revealed good facial proportions with a convex profile. The upper dental midline was shifted to the right 1.5 mm relative to the facial midline.

**Figure 1:**
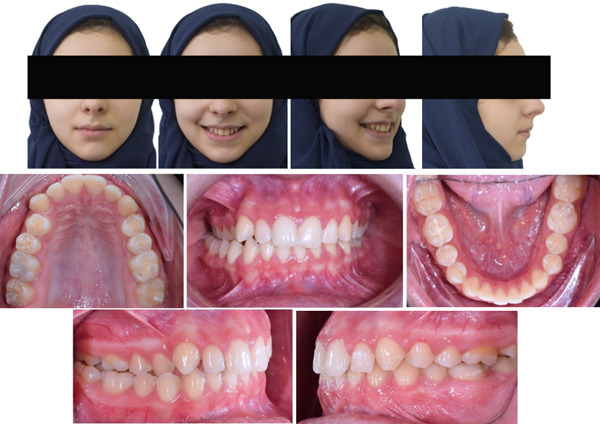
Pretreatment intraoral and extraoral photographs.

The patient had a Class I molar and Class II canine relationship on the right side and a Class I molar and canine relationship on the left side, with a 50% overbite and 4 mm overjet. A unilateral posterior crossbite was found on the right side due to unilateral constriction of the maxillary arch (Figure 1).

**Figure 2:**
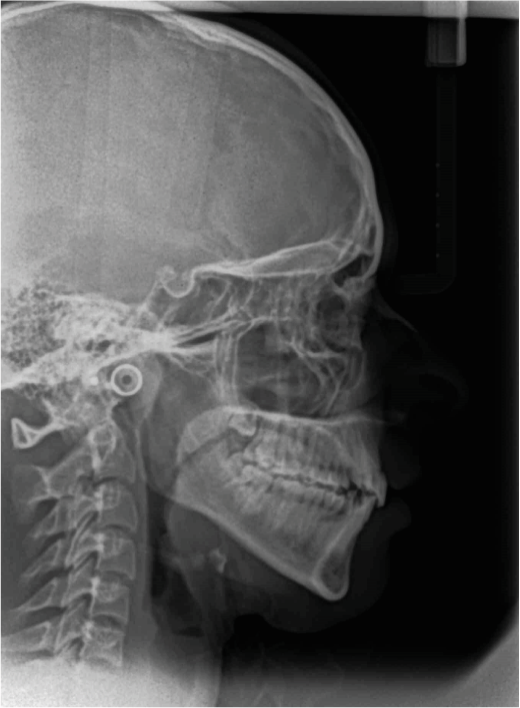
Pretreatment cephalometric radiograph.

**Figure 3:**
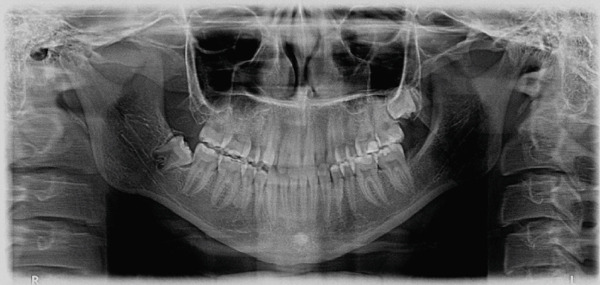
Pretreatment panoramic radiograph.

Radiographically, the patient had a Class I skeletal relationship, with an increased mandibular plane angle and lower anterior facial height. The upper and lower incisors were normally inclined (Table 1 and Figure 2). The upper right and lower left third molars were congenitally missing (Figure 3).

**Table 1:** Pretreatment cephalometric measurements.

**Area of study**	**Measurement**	**Mean**	**Pre-treat.**
Sagittal relationship	SNA	82^°^ ± 2°	83°
	SNB	80^°^ ± 2°	82°
	ANB	2^°^ ± 2°	1°
	NA–APg.	0^°^ ± 5°	4°
	Wits appraisal	1.2 ± 1.9 mm	1 mm

Vertical relationship	Mand. plane to FH	25^°^ ± 5°	28°
	Mand. plane to SN	32^°^ ± 5.1°	34°
	Max. plane to SN	8^°^ ± 3°	8°
	Max. to mand. plane	25^°^ ± 3°	28°
	*Y* axis SGn./SN	59^°^ ± 4°	60°
	Lower face height	55*%* ± 3*%*	0.56

Dental relationship	U inc. to max. plane	110^°^ ± 6°	103°
	U inc. to SN.	104^°^ ± 2°	100°
	U inc. to NA	22^°^ ± 5°	20°
	U inc. to NA (mm)	4 ± 3 mm	3 mm
	U inc. to L inc.	131^°^ ± 5°	130°
	L inc. to mand.	93^°^ ± 6°	91°
	L inc. to NB	25^°^ ± 6°	23°
	L inc. to NB (mm)	4 ± 2 mm	3 mm
	L inc. to APog. (mm)	1 ± 2 mm	1 mm

Soft tissue relationship	Upper lip to E‐line	−4 ± 2 mm	−3 mm
	Lower lip to E‐line	−2 ± 2 mm	−1 mm
	Nasolabial angle	100^°^ ± 10°	115°

### 2.2. Treatment Alternatives

Based on treatment objectives, the following options were discussed:

1. MARPE: A less invasive alternative to SARPE using skeletal anchorage. However, it still tends to expand on both sides and may not be ideal in true unilateral cases.
2. SARPE: An effective option for transverse discrepancies in skeletally mature patients, despite being more invasive, it requires hospitalization, general anesthesia, and has higher morbidity.
3. Corticotomy‐assisted expansion: A minimally invasive surgical technique that selectively reduces bone resistance on the crossbite side. It allows for differential expansion and more controlled tooth movement, making it a suitable option for unilateral posterior crossbite in adults.
4. Conventional orthodontic expansion: Using appliances like a quad‐helix or hyrax. However, this approach may lead to undesirable bilateral expansion in unilateral cases, potentially overcorrecting the unaffected side and causing more dental tipping.

The advantages and disadvantages of each option were discussed, and the patient chose option three as it was considered less invasive and less costly (service available free in the center), and informed consent was signed.

### 2.3. Treatment Objectives

- Resolve the unilateral posterior crossbite.
- Correct upper dental midline deviation.
- Achieve functional occlusion with maximum intercuspation, with normal overbite and overjet.
- Achieve a Class I canine relationship on the right side and maintain existing Class I on the left side.

The treatment plan was chosen to achieve the treatment objectives through corticotomy‐assisted expansion with fixed orthodontic appliances.

## 3. Treatment Progress

A corticotomy was performed on the buccal side of the right maxillary segment (Figure 4). After administration of local anesthesia, an intrasulcular incision was made buccally and vertically. Single‐flap corticotomy (SFC) with the papilla preservation technique principle was used. In brief, only the teeth that required the greatest amount of movement were included in the segmental design of the procedure. Therefore, the extent of the horizontal, intrasulcular incision was limited to the area distal to the upper right canine and to the distal surface of the upper right second molar. A full‐thickness mucoperiosteal flap was elevated to expose the underlying alveolar bone. Since the cortical plate thickness was > 2 mm, corticotomy was performed without grafting. Analgesics and antibiotics were also prescribed. Homecare instructions were provided, including the use of a chlorhexidine rinse.

Figure 4Surgical procedure of corticotomy. (a) An intrasulcular incision was made buccally and vertically. (b) Full‐thickness (single‐flap corticotomy [SFC] with papilla preservation technique) was reflected. (c) Selective alveolar decortication lines and points are made. (d) The flap is sutured back.

**Figure:**
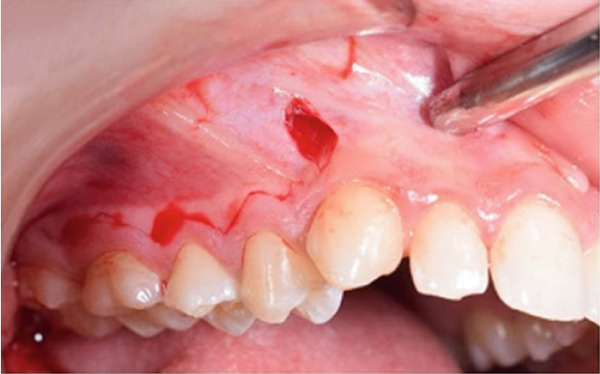
(a)

**Figure:**
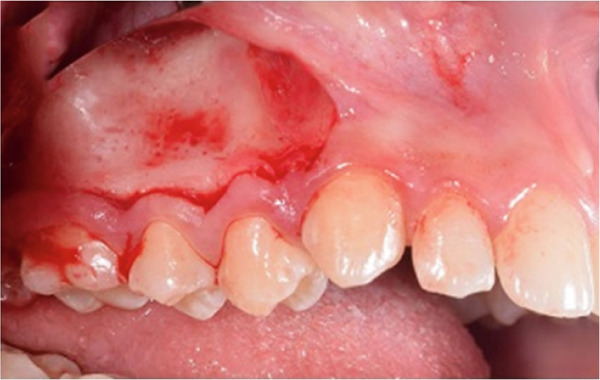
(b)

**Figure:**
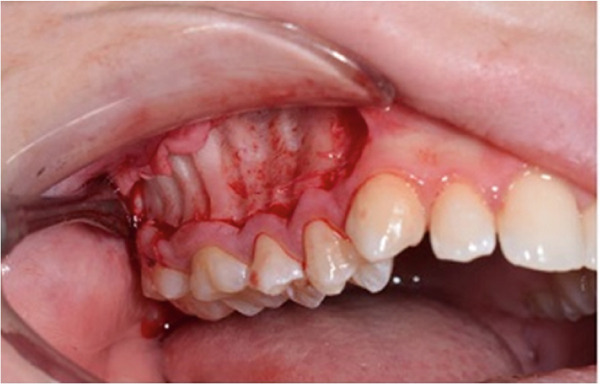
(c)

**Figure:**
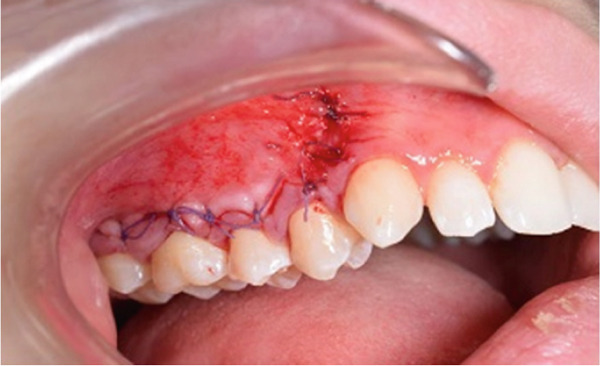
(d)

**Figure 5:**
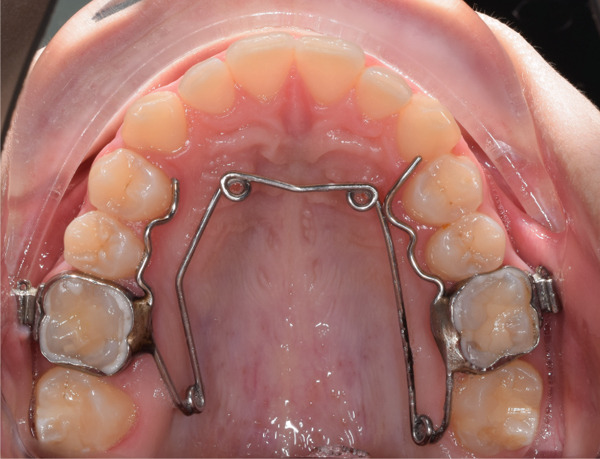
Quad‐helix appliance.

Ten days postcorticotomy, expansion was initiated using a quad‐helix appliance (Figure 5) until overcorrection was achieved after 2 months. For retention, the appliance was left in place for 3 months.

The upper and lower teeth were bonded with preadjusted edgewise fixed orthodontic appliances (Gemini stainless steel metal brackets; 3M Unitek, Monrovia, CA, United States) using a Roth prescription (0.022‐in. slot).

Leveling and alignment were carried out using a full archwire sequence starting with 0.014‐ and 0.016‐in. nickel‐titanium (Niti) wires, followed by 0.016 × 0.022‐in. and 0.017 × 0.025‐in. Niti wires. This was progressed to 0.017 × 0.025‐in. and 0.019 × 0.025‐in. stainless steel wires for space closure, torque control, and the use of Class II elastic to achieve Class I canine on the right side.

**Figure 6:**
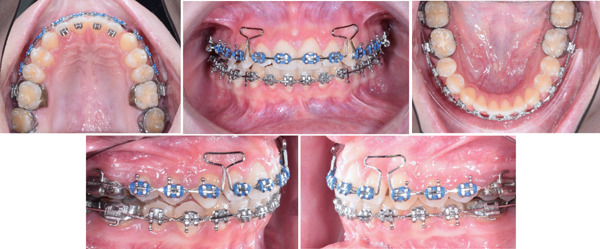
Mid‐treatment progress.

Final detailing was achieved with 0.019 × 0.025‐in. stainless steel arch wires Figure 6.

The arches were coordinated to achieve proper alignment, leveling, and occlusal stability, and the retention plan involved an upper Hawley retainer and a lower fixed retainer from canine to canine.

## 4. Results

**Figure 7:**
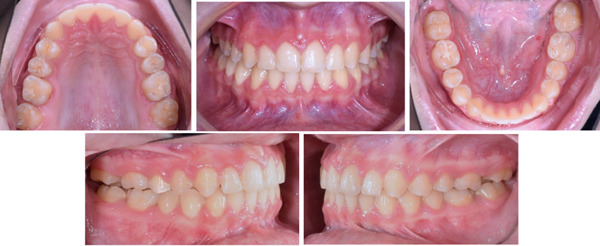
Posttreatment intraoral photographs.

**Figure 8:**
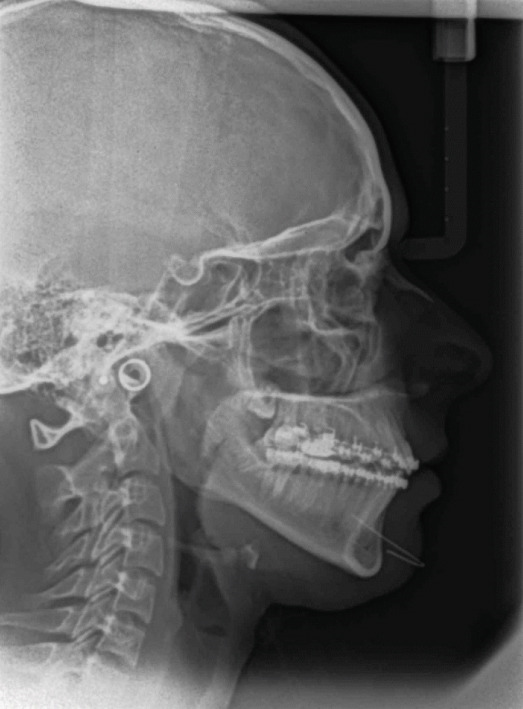
Posttreatment cephalometric radiograph.

**Figure 9:**
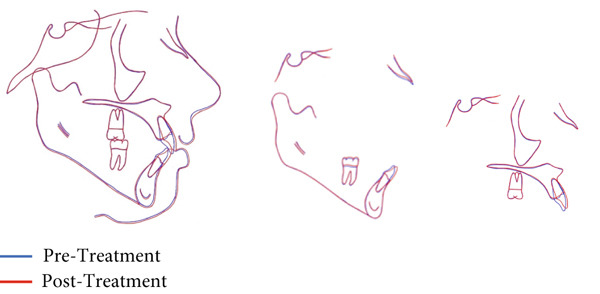
Overall and regional superimposition.

Treatment was performed for 12 months. The crossbite was corrected, and a normal overbite and overjet were achieved with Class I canine and molar relationships (Figure 7). The intermolar and intercanine distances increased by 5 and 3 mm, respectively. Cephalometric analysis revealed no significant changes, and superimposition showed no significant skeletal changes (Table 2 and Figures 8 and 9). The periodontal examination, conducted through standard clinical procedures such as visual inspection, measurement of probing depths, and assessment of gingival health, revealed no abnormalities.

**Table 2:** Posttreatment cephalometric measurements.

**Area of study**	**Measurement**	**Mean**	**Post-treat.**
Sagittal relationship	SNA	82^°^ ± 2^°^	83°
	SNB	80^°^ ± 2^°^	82°
	ANB	2^°^ ± 2^°^	1°
	NA–APg.	0^°^ ± 5^°^	5°
	Wits appraisal	1.2 ± 1.9 mm	1 mm

Vertical relationship	Mand. plane to FH	25^°^ ± 5^°^	30°
	Mand. plane to SN	32^°^ ± 5.1^°^	35°
	Max. plane to SN	8^°^ ± 3^°^	8°
	Max. to mand. plane	25^°^ ± 3^°^	29°
	*Y* axis SGn./SN	59^°^ ± 4^°^	60°
	Lower face height	55*%* ± 3*%*	0.57

Dental relationship	U inc. to max. plane	110^°^ ± 6^°^	107°
	U inc. to SN	104^°^ ± 2^°^	105°
	U inc. to NA	22^°^ ± 5^°^	23°
	U inc. to NA (mm)	4 ± 3 mm	5 mm
	U inc. to L inc.	131^°^ ± 5^°^	125°
	L inc. to mand.	93^°^ ± 6^°^	92°
	L inc. to NB	25^°^ ± 6^°^	25°
	L inc. to NB (mm)	4 ± 2 mm	4 mm
	L inc. to APog. (mm)	1 ± 2 mm	3 mm

Soft tissue relationship	Upper lip to E‐line	−4 ± 2 mm	−2 mm
	Lower lip to E‐line	−2 ± 2 mm	−1 mm
	Nasolabial angle	100^°^ ± 10^°^	113°

## 5. Discussions

In skeletally mature patients, the treatment of maxillary transverse discrepancies can be challenging and complicated [12]. Previous reports have suggested corticotomy‐assisted maxillary expansion as an alternative to SARPE for unilateral posterior crossbite in skeletally mature patients [2, 12, 13].

Corticotomy was performed only on the crossbite side to overcome unnecessary contralateral expansion, accelerate tooth movement, and encourage more tissue turnover. Therefore, expansion occurs faster on the crossbite side than on the normal side [2]. Thus, it reduces unnecessary expansion on the other side. The duration of crossbite correction is relatively shorter (8 weeks), without unnecessary expansion on the other side, which is considered an additional advantage of the technique, as reported in a previous case report [2] and could be attributed to less expansion on the other side as less time for teeth to move in comparison to the corticotomy side, where teeth move faster.

**Figure 10:**
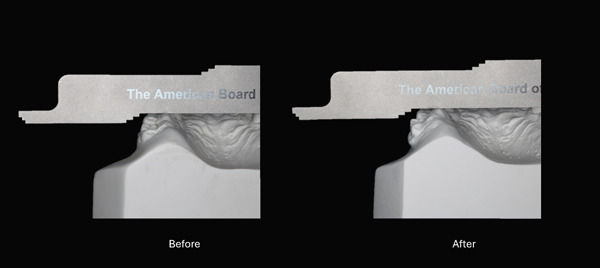
Using the ABO gauge for the study cast before and after treatment.

Using an American Board of Orthodontics (ABO) objective grading system gauge, the buccal and palatal cusps of the first molars and premolars were measured before and after (Figure 10) and found to be within the normal range to the transverse plane. This indicates that the expansion achieved was a result of bodily movement of the teeth rather than dental tipping, unlike the conventional methods of expansion for skeletally mature patients, where the expansion is expected to be more due to buccal tipping [14].

**Figure 11:**
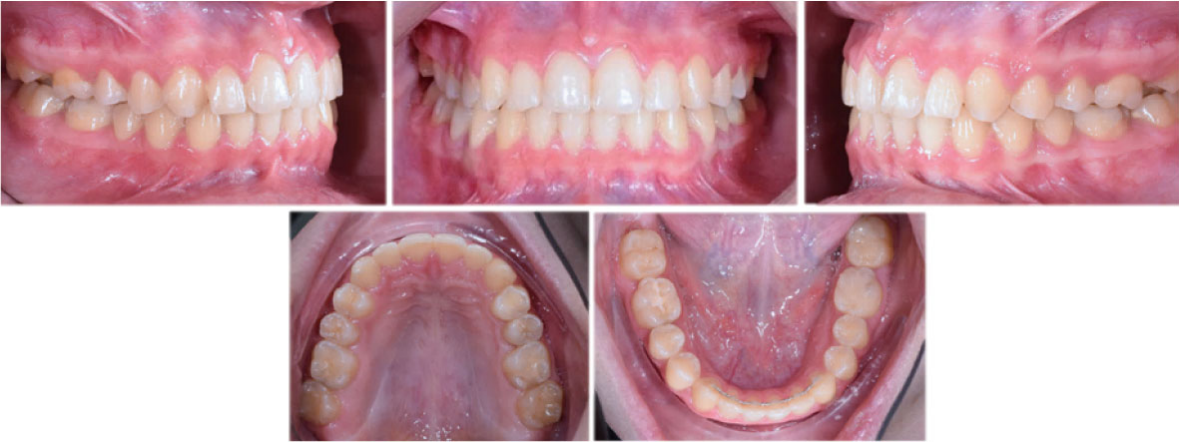
Follow‐up after 1 year.

In this case, periodontal health was maintained without any complications; similar findings were observed by Hassan et al. [2] and Silva‐Coll et al. [13]. After 1 year of retention, the occlusion was stable (Figure 11).

Few clinical studies have been published in the context of corticotomy‐assisted maxillary arch expansion. One case report published corticotomy‐assisted rapid maxillary arch expansion without ridge augmentation in a 14‐year‐old girl [15]. However, in adult patients, this was reported by Hassan et al. [2], and the efficacy of this approach and documented stable long‐term results for 7 years was reported by Silva‐Coll et al. [13].

MARPE has gained recognition as a feasible alternative to SARPE for the treatment of maxillary transverse deficiency in skeletally mature individuals. A recent study by Nguyen et al. [16] presented a case report of a 27‐year‐old female patient with bilateral posterior crossbite, demonstrating a 94% success rate, which makes it very successful in the treatment of bilateral maxillary expansion, but for unilateral posterior crossbite, it is unclear how successful it is. In this context, selective alveolar bone corticotomy is identified as a less invasive option for unilateral posterior crossbite when compared to SARPE, as it involves periodontal surgery with reduced postoperative complications, such as soft tissue edema and mild pain. Economically, it is considered a more cost‐effective solution, especially for skeletally mature patients with unilateral posterior crossbite rather than bilateral crossbite.

**Figure 12:**
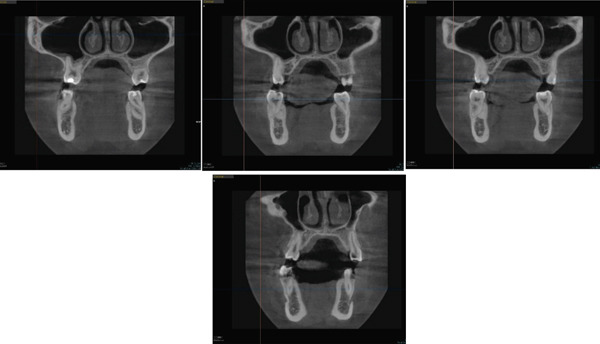
A CBCT 2 years posttreatment.

An important limitation of this study is the absence of pretreatment CBCT, which restricts the ability to directly quantify skeletal changes associated with the buccal‐only corticotomy. Nevertheless, a CBCT taken 2 years posttreatment (Figure 12) demonstrated adequate bone volume and stability in the expanded region. This finding suggests that the procedure did not compromise alveolar bone support and may have contributed to the long‐term maintenance of the achieved expansion. Similar observations have been reported in previous studies [2, 13], where corticotomy‐assisted expansion was evaluated using conventional radiographs, dental casts, or posttreatment imaging rather than baseline CBCT. These reports consistently indicate that corticotomy‐assisted approaches can achieve stable transverse expansion without adverse skeletal consequences. Therefore, while the absence of baseline CBCT is acknowledged as a limitation, the current findings, supported by long‐term follow‐up imaging, add to the body of evidence suggesting that buccal‐only corticotomy may provide stable outcomes with sufficient bone support. Future prospective studies employing both pre‐ and posttreatment CBCT are warranted to further delineate the skeletal and dental contributions of this technique.

Posttreatment periodontal health was evaluated clinically through routine periodontal examination, including probing depth, gingival condition, and mobility, and no pathological findings were observed. However, more quantitative periodontal parameters could further validate soft tissue stability, which may be considered in future studies.

## 6. Conclusions

In the presented case, unilateral corticotomy‐assisted maxillary arch expansion was effective in treating true unilateral crossbite in skeletally mature patients. This approach may provide an efficient treatment option for unilateral maxillary transverse deficiencies in adults.

## Disclosure

The authors confirm that they all meet the criteria for authorship and have approved the final version of the manuscript.

## Conflicts of Interest

The authors declare no conflicts of interest.

## Author Contributions

**Ahmad A. Al-Fraidi:** conceptualization, literature review, methodology, original draft preparation. **Rana S. Alamri:** conceptualization, literature review, methodology, original draft preparation. **Baker M. Sedqi:** conceptualization, literature review, methodology, original draft preparation.

## Funding

No funding was received for this manuscript.
